# Supplementary material for: Identification of photoperiod-induced specific miRNAs in the adrenal glands of Sunite sheep (Ovis aries)
Source: Front Vet Sci. 2022 Jul 22;9:888207. doi: 10.3389/fvets.2022.888207 (PMC9354845; doi:10.3389/fvets.2022.888207)
Supplement: Supplementary file 1 [file Data_Sheet_1.ZIP › Supplementary file/miRDeep2 file.zip!/miRDeep2 file/Novel_Structure/SP42A2/Novel_160.pdf]

Provisional ID	:	NC_019459.2_6467
Score total	:	90.7
Score for star read(s)	:	3.9
Score for read counts	:	83.7
Score for mfe	:	1.4
Score for randfold	:	1.6
Score for cons. seed	:	
Total read count	:	176
Mature read count	:	174
Loop read count	:	0
Star read count	:	2

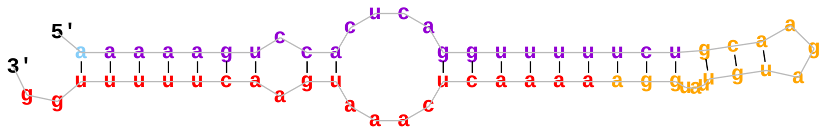

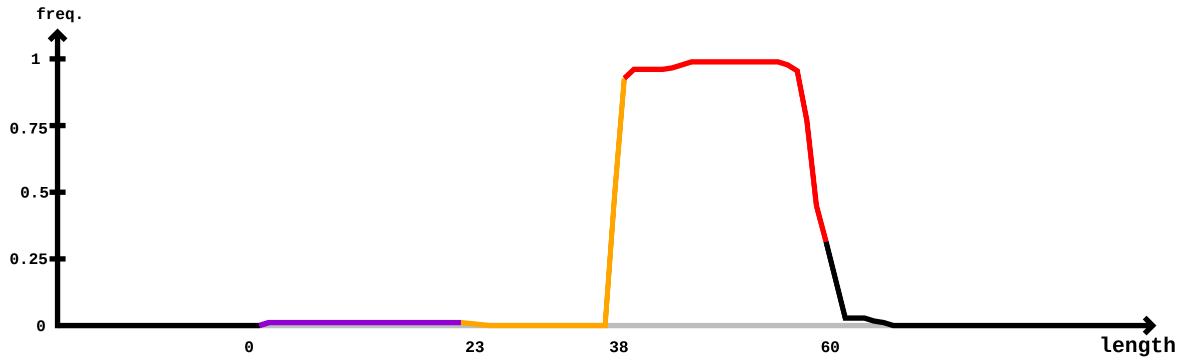

Star **Mature**

```
5'- uauaauauaucagguuggccaaaaaguccacucagguuuuucugcaagauguuauggaaaaacucaaaugaacuuuuuggccaacucaauauuuuucuugggguugacucacu  -3'   obs
    uauaauauaucagguuggccaaaaaguccacucagguuuuucugcaagauguuauggaaaaacucaaaugaacuuuuuggccaacucaauauuuuucuugggguugacucacu         exp
    ....(((((..((((((((((((((.((....(((((((((((...)))...))))))))....)).))))))))))))))..))))).....((((.....))))..   reads   mm   sample
    .....................aaaaguUcacucagguuuuucu......................................................................   1   1   seq
    .....................aaaaguccacucagguuuuucu......................................................................   1   0   seq
    .........................................................aaaaacucaaaugaacuu......................................   1   0   seq
    .........................................................aaaaacCcaaaugaacuu......................................   1   1   seq
    .........................................................aaaaacucaGaugaacuuu.....................................   1   1   seq
    .........................................................aaaaacucaaaugaacuuu.....................................   3   0   seq
    .........................................................aaaaacCcaaaugaacuuuu....................................   1   1   seq
    .........................................................aGaaacucaaaugaacuuuu....................................   1   1   seq
    .........................................................aaaaacucaaaugaacuuuu....................................   30  0   seq
    .........................................................aaaaacucaaaugaacuuuG....................................   1   1   seq
    .........................................................aaaaGcucaaaugaacuuuuu...................................   1   1   seq
    .........................................................aaaaacucaGaugaacuuuuu...................................   5   1   seq
    .........................................................aaaaacucaaaugaacuuuuA...................................   36  1   seq
    .........................................................aaaaacucaaaugaacuuuuug..................................   5   0   seq
    .........................................................aaaaacucaaaugaacuuuuuA..................................   1   1   seq
    .........................................................aaaaacucaaGugaacuuuuug..................................   1   1   seq
    ..........................................................aaaacucaGaugaacuuuuu...................................   6   1   seq
    ..........................................................aaaacCcaaaugaacuuuuu...................................   9   1   seq
    ..........................................................aaaacCcaaaugaacuuuuug..................................   16  1   seq
    ..........................................................aaaacucaaaugaacuuuuugg.................................   2   0   seq
    ..........................................................aaaacucaGaugaacuuuuugg.................................   43  1   seq
    ..........................................................aaaacucaaaugaacuuuuuggA................................   1   1   seq
    ...........................................................aaacucaGaugaacuuuuug..................................   1   1   seq
    ...........................................................aaacucaaaugaacuuuuuggU................................   2   1   seq
    ...........................................................aaacAcaaaugaacuuuuuggc................................   2   1   seq
    ...........................................................aaacCcaaaugaacuuuuuggc................................   1   1   seq
    ...............................................................ucaaaugaacuuuuuggccaU.............................   1   1   seq
    ................................................................caaaugaacuuuCuggccaa.............................   1   1   seq
    ................................................................Uaaaugaacuuuuuggccaac............................   1   1   seq
    .................................................................aaaugaacuuuuuggccaacu...........................   2   0   seq
```
